# Recommendations for the analysis of leading indicators in epidemic surveillance

**DOI:** 10.1371/journal.pdig.0001759

**Published:** 2026-10-05

**Authors:** Jonathon Mellor, Maria Tang, Robert S. Paton, Thomas Ward

**Affiliations:** Chief Data Officer Group, UK Health Security Agency, London, United Kingdom; The University of Sheffield, UNITED KINGDOM OF GREAT BRITAIN AND NORTHERN IRELAND

## Abstract

Leading indicator analyses, a type of time series analysis, are frequently used to assess how epidemic surveillance signals relate to one another. Strong leading indicators can drive early, and better decisions in public health systems. We conducted a narrative review of leading indicator studies and evaluated methodological limitations in their reporting and analysis, which we used to generate recommendations in retrospective surveillance analyses. Achievable recommendations are provided based on the reporting and analysis components of real-time epidemic time series analysis. We provide contextual examples from the literature of previous studies demonstrating good practice and highlighting areas for improvement. We present a checklist and workflow that emphases real-time constraints. These recommendations include causal mechanism reporting, sample coverage and bias, reporting delays and data revisions, multiple event measurement, spatio-temporal granularly, time varying relationships, smoothing, transformations, uncertainty, and predictive utility. By following these good practices, analysts can improve research on leading indicators to make analyses more directly actionable for public health decision makers. This work builds on the existing methodology literature by giving a broader framework for overall good practices.

## Introduction

The effective mitigation of infectious disease spread, and healthcare resource management often relies on partially complete information. Many infections are unobserved, and those cases detected are subject to severity biases, biological and reporting time delays. During high consequence outbreaks testing capacity can be exceeded and alternative signals become essential to determine trends. High-quality reliable population-level signals may not be available in real-time, and when available, may be understood too late to make effective policy or operational decisions. Data sources which reliably indicate future infection trends can support decision making, improve the timing of interventions and communicate risk [1]. We refer to a key surveillance data source, such as hospital admissions with a disease as the “signal”, and another data source which may give an early indication of the signal as the “indicator” going forward. The leading indicator is said to lead the signal if it has a strong relationship (such as a correlation) at some negative lag time shift, in that the indicator value precedes the signal.

The SARS-CoV-2 pandemic led to a proliferation of new surveillance data sources to investigate for a range of different pathogens and transmission routes. Indicators are often not traditional epidemiological surveillance (e.g., cases, admissions, deaths), and instead include other collection methods such as internet-based [2], app-based [3] and wastewater detection [4]. These indicators are often opportunistically captured, or administrative data collected for alternative purposes.

While indicators may often be available more rapidly than traditional surveillance, they are subject to different, and often more substantial biases. “Leading indicators”, often called “early warning signals” [5] or “predictors”, are widely analysed in epidemiology and play an important role in validating surveillance systems [6,7]. Typically, the utility of a leading indicator for a given signal will be demonstrated by statistically evaluating the agreement between the two time series at different lead times. Early warning indicators are frequently harnessed to provide forecasts of signals [8,9]. Understanding indicator/signal relationships is both useful in outbreaks [10], emerging pathogen situations [11] and established disease systems [12]. There are multiple reviews in the literature exploring epidemic time series relationships including for influenza [13], gastrointestinal syndromic surveillance [14], and vector borne disease indicators [15].

However, not all leads between indicator and signal are the same. Leads can be:

- An ***association*** lead—where retrospective evaluations find an alignment between indicator and signal.
- A ***predictive*** lead—the indicator improves out-of-sample performance of a forecast model for the signal at some future time point.
- An ***operational*** lead—a predictive lead which accounts for data latency, reporting delays and real-time factors.

Many retrospective evaluations of leading indicators focus on establishing an association lead, but this can be challenging to translate into real-time utility.

The utility of a leading indicator is inherently based on real-time use, providing decision makers an information edge beyond the signal alone. However, thorough evaluation of leading indicator utility is naturally retrospective. The retrospective analysis of leading indicators can often cause overoptimistic reporting of utility, or present results which are detached from real-world use cases. While there is a diverse range of methods for evaluating leading indicators [16], there is not currently a clear set of guidance for practitioners to consider when designing analyses in epidemic contexts. Current approaches for estimating lead times range from advanced statistical modelling [17] to visual inspection [18,19] highlighting the need for guidance. This mismatch between real-world application and the retrospective methods used to evaluate leading indicators is not inevitable, and by following good practices the conclusions intended for public health action can become more reliable. In this work, we will outline key recommendations for the reporting and analyses of leading indicators for epidemiological surveillance systems.

Throughout this article, we present supporting figures demonstrating key concepts of the recommendations. The figures are created via simulation of plausible epidemic data, with the methods and processing outlined in Supplementary Section 1 in S1 Appendix.

## Recommendations

We present ten recommendations for researchers to consider when conducting temporal analysis of epidemiological data, particularly leading indicator analyses. These are summarised in Table 1, with further detail provided in each section.

**Table 1:** Recommendation checklist to assess operational utility of leading indicator analyses with examples from the literature.

Type	Number	Name	Description	Examples from literature
Reporting	1	Reporting of causal mechanisms	Researchers should articulate how the indicator and signal relate mechanistically, and why a leading relationship is expected.	Dolan and colleagues [20], van Asten and colleagues [21]
	2	Sample coverage and bias	Researchers should explain how the populations the signal and indicator draw from overlap with each other.	Morvan and colleagues [22], Kuster and colleagues [23]
	3	Reporting delays and data revisions	Researchers should clearly state the reporting delays the indicator and signal are subject to in real time, along with the extent of retrospective revisions.	Reinhard and colleagues [24], Peccia and colleagues [25], Grover and colleagues [26]
	4	Single or multiple events	Researchers should articulate whether a subject can influence the indicator or signal once or at multiple time points.	Wood [27], D’Auost and colleagues [28]
Analysis	5	Temporal and spatial granularity	Researchers should avoid generating unmeasured data and prioritise the temporal and spatial scale of the signal.	Shi and colleagues [29], Armenta-Castro and colleagues [30]
	6	Time-varying relationships	Researchers should present the evaluation over multiple time periods to demonstrate if leading relationships are consistent.	Anderson and colleagues [31], Cook and colleagues [32]
	7	Smoothing and denoising	If smoothing is necessary, researchers should choose a consistent method for avoiding the use of information unavailable in real-time.	You and colleagues [33], Morbey and colleagues [34], Steyn and colleagues [35,36]
	8	Transformation: logs, rates, scaling, magnitude and direction	Researchers should mathematically transform their data appropriate to the infection process and sampling method of each data, and align the data with the decision-making process.	Rø and colleagues [17], McGough and colleagues [37]
	9	Uncertainty	Researchers should propagate uncertainty through their analyses to conclusions about temporal relationships.	Asplin and colleagues [38], Ammerman and colleagues [39]
	10	Predictive utility out of sample and baselining	Researchers should demonstrate indicator lead times through predictive modelling of the signal and compare to a baseline.	Reich and colleagues [8], McDonald and colleagues [40]

## Recommendations: Reporting

### 1. Reporting of causal mechanisms

*Researchers should articulate how the indicator and signal relate mechanistically, and why a leading relationship is expected.*

Good leading indicators are not useful because of coincidence. Practitioners should understand how the indicator and signal relate, and the common factors between both. While some indicator-signal relationships may be intuitive, explicitly stating (preferably with a diagram), how the indicator-signal directly or indirectly relate allows readers to have a mental model of the processes involved. More complex relationships have more biases and uncertainty involved [41], which can transparently be interrogated when made explicit. If the analyst does not know the causal link between indicator and signal, then this is a good opportunity to work with those who can investigate. Diagrams of causal relationships are frequently used in the development of compartmental models, which helps articulate to the reader the researchers’ assumptions analysis [42].

Furthermore, from this causal relationship there should be a clear articulation of why, mechanistically, the researchers believe there may or may not be a leading relationship between indicator and signal [43]. In Fig 1 we show explicitly why we think that a Google search might lead in time a hospital admission at the individual level, which helps us infer population-level trends. The use of a Directed Acyclical Graph (DAG) can help articulate cause-and-effect hypotheses clearly [44,45]. The exploration of causal mechanism helps establish based on more than association between signals.

**Fig 1:**
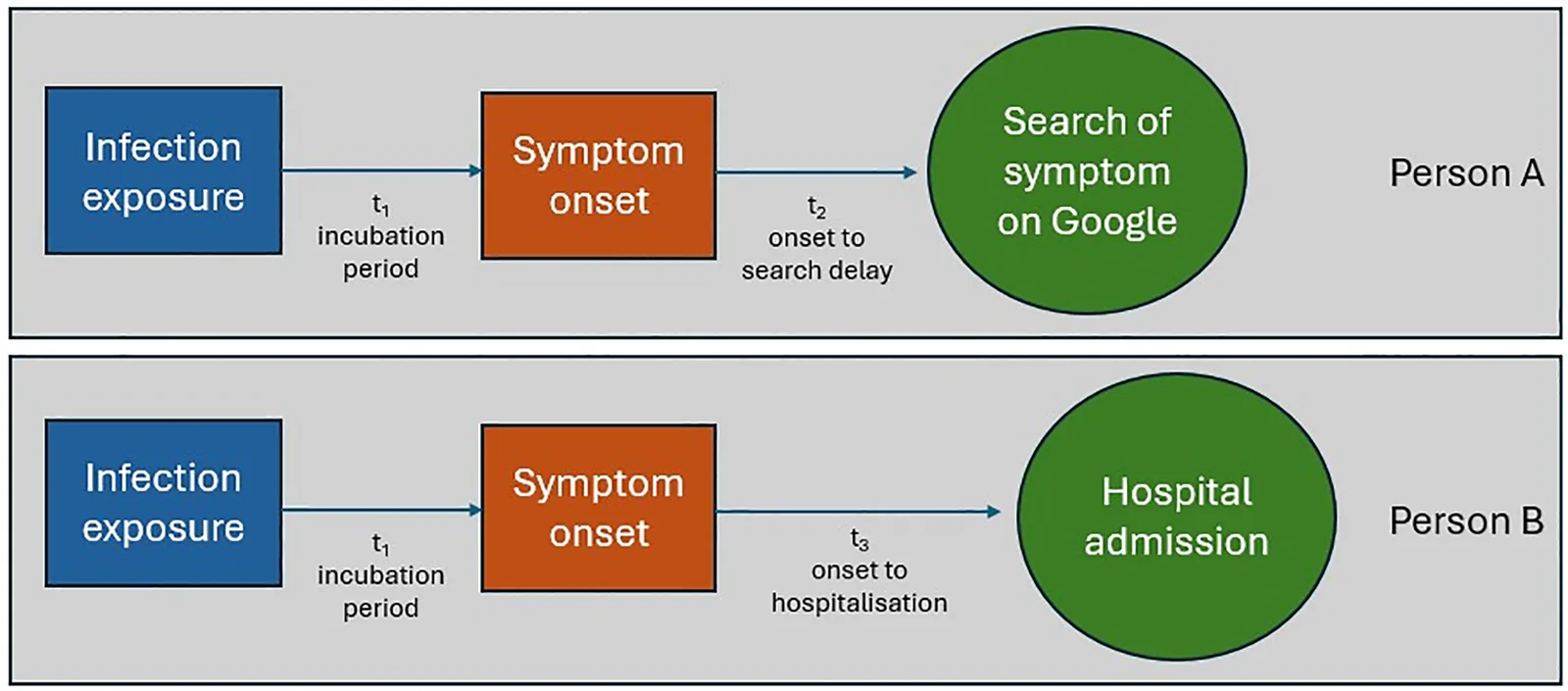
Example Directed Acyclical Graph (DAG) showing the individual-level causal chain of events for observations of Google symptom search and hospital admission counts. By showing the different time delays involved at an individual level, we can propose what the leading indicator relationship may be driven by. In this example if *t*_2_ < *t*_3_ on average, *t*hen in a simple epidemic, we would expect counts of Google symptom searches to lead hospital admission counts by approximately *t*_3_–*t*_2_.

A focus on mechanism can help untangle why lead times are observed, lending credibility to claims. An example of where this articulation of mechanism strengthens understanding is in Dolan and colleagues [20]. The authors investigate the use of non-prescription medication sales to improve respiratory-associated death forecasts. They propose that sales data are proxies for community risk which precedes infection for individuals who later die, clearly articulating the mechanism at play.

In van Asten and colleagues [21] the authors explore the timing between influenza A and other respiratory virus infections occurrences. The researchers explicitly state that they are exploring the relationship between influenza wave timing and the timing of other viruses and provide hypotheses for why this might occur including biological inhibition and surveillance changes. This helps to ground the time series analyses in mechanism—for example, they highlight that enteric viruses have a weaker identified relationship than respiratory infections.

### 2. Sample coverage and bias

*Researchers should explain how the populations the signal and indicator draw from overlap with each other.*

Rarely are the signal and indicator data drawn from the exact same population, which would reduce amount of bias in their comparison. Instead, the indicator may be from a sub-group or different cohort than the signal population. As a result, lead times and utility may be driven by the differences between cohorts, rather than biological or reporting factors. As we expect infection rates to vary in sub-groups of the population, such as by age, sex and location [46,47], the surveillance of different sub-groups can generate a leading relationship between indicator and signal.

Furthermore, different sub-groups in a population will have different propensities to engage with measurement for surveillance data. For example, younger individuals may be more likely to engage with online surveillance systems [48], and those more severely impacted by infection are more likely to engage in healthcare-seeking behaviour [49]. However, in cases where there are unclear relationships between the indicator and signal population, such as non-overlapping geographies, this makes the causal link between the two data more indirect, undermining the leading indicator strength of evidence. Reporting the cohort characteristics of both the indicator and signal sample population, particularly demographic and spatial biases, helps to explain the relationship between surveillance data. Fig 2 shows that there can be clear differences in populations based on the geography covered by different data, and that populations are not guaranteed to overlap between data.

**Fig 2:**
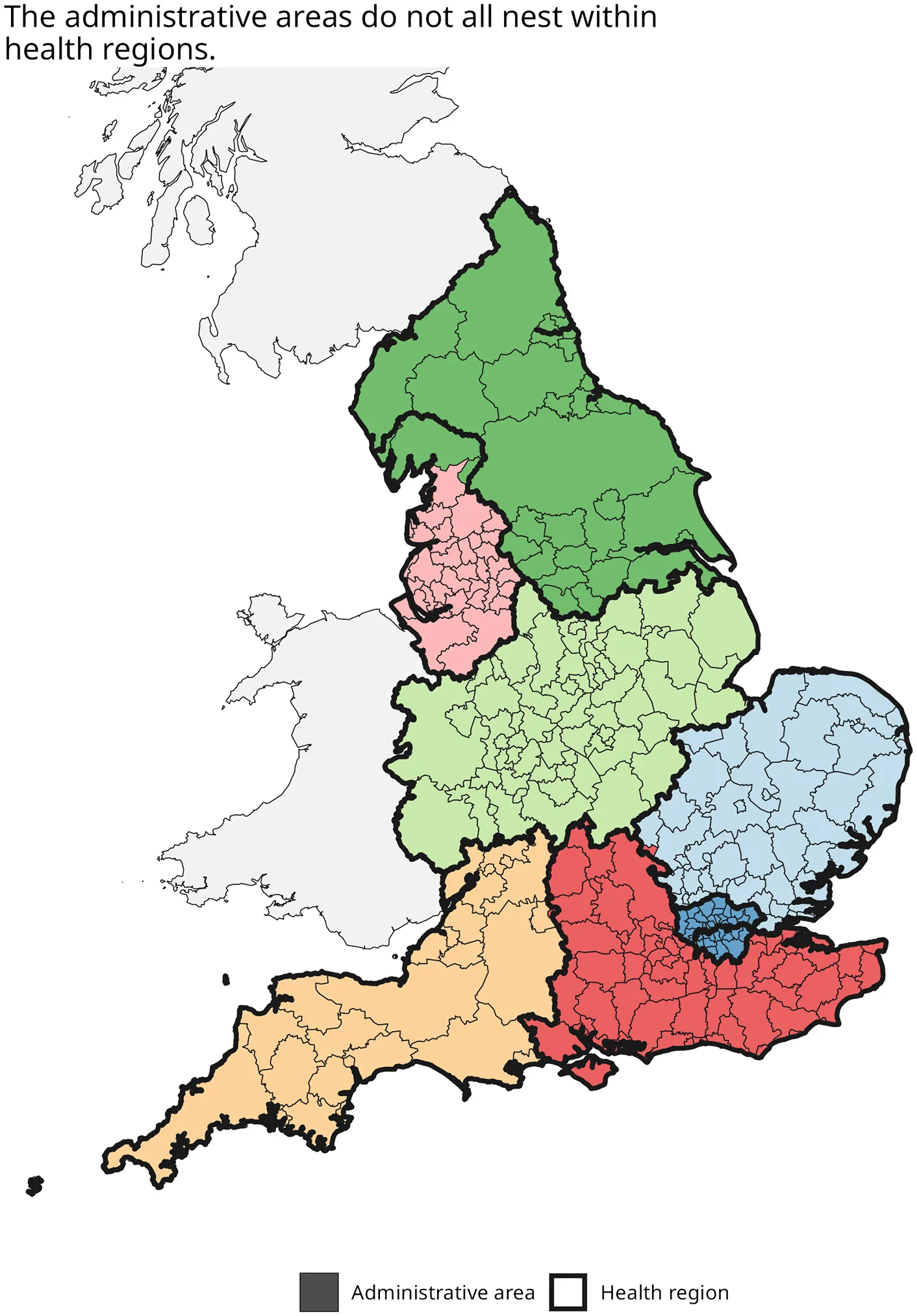
Map of England with healthcare regions (solid black lines) and administrative areas (coloured by the healthcare region their centre resides) highlighted. Data could be gathered at an administrative or a healthcare region, which may not overlap perfectly—highlighting the importance of describing the populations of the indicator and signal. Data processed and figure generated with R, using spatial boundaries. Source: Office for National Statistics licensed under the Open Government Licence v.3.0. Contains OS data Crown copyright and database right 2026. Administrative boundaries: https://geoportal.statistics.gov.uk/datasets/ons::local-authority-districts-december-2024-boundaries-uk-bfc-2/about. Healthcare boundaries: https://geoportal.statistics.gov.uk/datasets/ons::nhs-england-regions-january-2024-boundaries-en-bfc/about.

Not only are individuals from different subgroups more or less likely to engage in surveillance measurement, but differential severity can also impact the timings of infection events [50]. Individuals who are more severely impacted by an infection will seek healthcare earlier than those with mild infections [51], further complicating the indicator and signal relationship.

Morvan and colleagues [22] explore wastewater as an indicator of SARS-CoV-2 community prevalence. The authors clearly state the difference between the wastewater catchment sites and regions used to estimate prevalence and clearly articulate the mapping process, helping the reader to understand this spatial limitation, and that different populations will contribute to each surveillance source.

In the example of Kuster and colleagues [23], the authors compare surveillance of influenza tests in Central Ontario (a region) to episodes of invasive pneumococcal disease in Toronto/Peel (sub-regions in Ontario). The non-matching locations of the study make drawing conclusions challenging in this analysis, influenza trends within sub-regions not covered by the invasive pneumococcal disease surveillance could be impacting the analysis. While having non-matching geographies does hinder analysis, it was important for the authors to state.

### 3. Reporting delays and data revisions

*Researchers should clearly state the reporting delays the indicator and signal are subject to in real time, along with the extent of retrospective revisions.*

Real-time surveillance systems must work with the data they receive, however, in retrospective analyses it is often more convenient to use finalised, revised or cleaned versions of data sets. Using finalised data, where reporting delays and backfilling data revisions are not present, biases results and can over-promise utility, producing a “best case” lead time. Practitioners cannot rely on consistent data quality, timeliness or completeness in real time, and therefore latency and the extent of data revisions are critical factors to report [52].

It is important to understand what information is available at what point in time, as delays and corrections can reduce estimated lead times and degrade indicator performance [53]. An indicator that takes one week to appear in surveillance systems but has a lead time relative to the signal of two weeks, will have a lead time of one week in practice as some of the lead time is lost to data latency. Similarly, an indicator that suffers from backfilling might only have a strong predictive correlation with the signal time series retrospectively.

Often, real-time individual-level epidemiological data, such as tests reported by collection date, can appear to decline in the most recent version of a time series [54]. This effect can be an artefact of reporting delays causing right truncation, rather than a true trend in the time series [55]. This incompleteness can be addressed through nowcasting, which adds uncertainty, or truncating the data until it is complete, adding latency and therefore reducing estimated lead times [56]. Alternatively, the data can be explored according to the date the information was made available for analysis – the report date – which is complete in real-time but induces a bias [57]. The difference between test date and report date is demonstrated via simulation in Fig 3.

**Fig 3:**
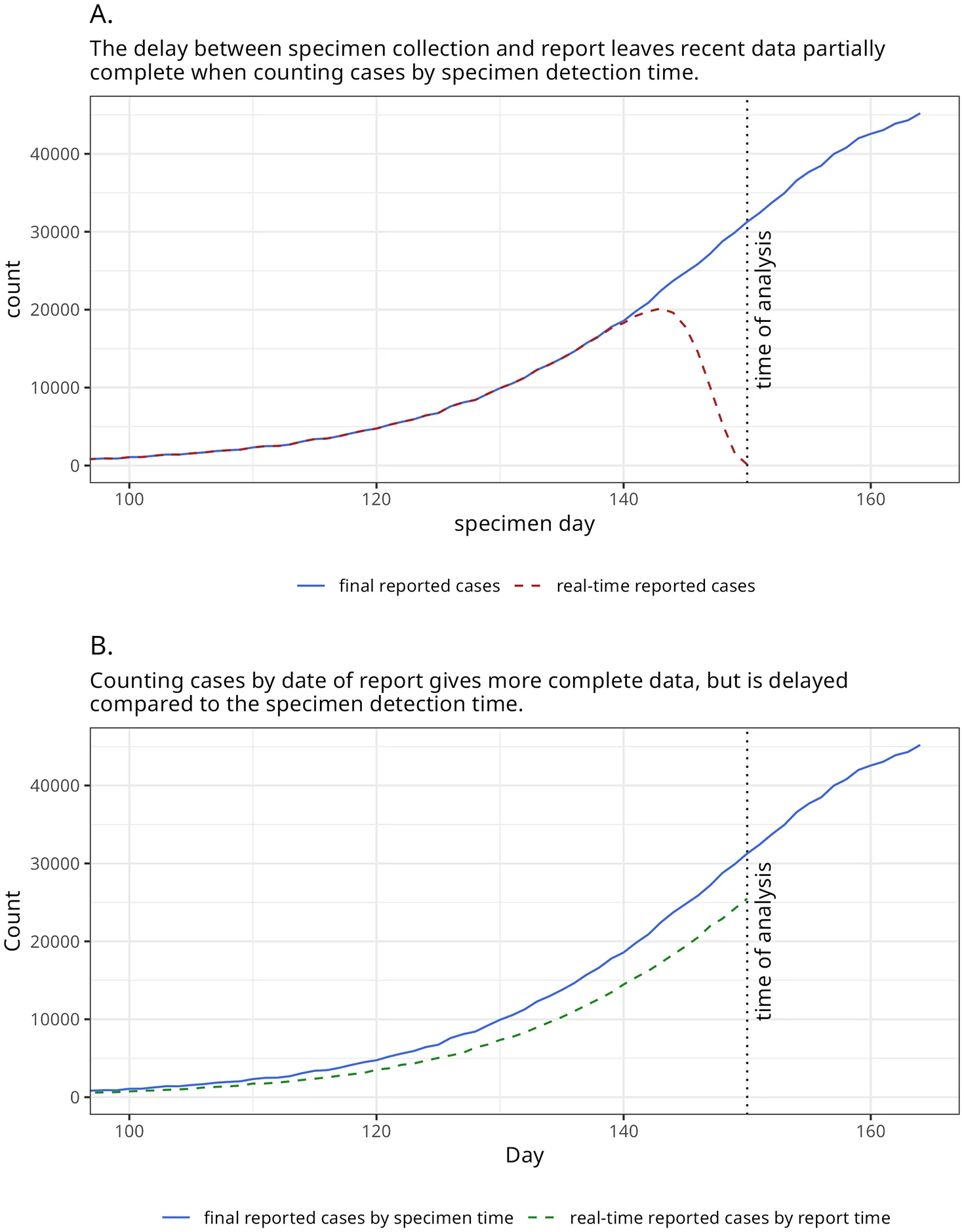
Using a simulation of epidemiological data, the effects of reporting delays are presented, comparing real-time versus final (retrospective) data availability and replicating an analysis from a line list. **(A)** shows that when indexed by a primary (specimen collection) time, then a real-time count will be partially complete due to delays. **(B)** demonstrates that presenting by the report time helps reduce the incompleteness, but the reporting delay compared to the final data by specimen date impacts the trend.

In the example of the COVIDcast API from Reinhart and colleagues [24], a collection of real-time indicators of COVID-19 in the United States, revisions were a key focus of the data collection, and this information was readily available for analysis in the form of an “issue date”. By surfacing this data revisions information better indicator analyses can be performed.

In the work of Peccia and colleagues [25], the authors perform a non-linear distributed lag analysis of SARS-CoV-2 wastewater RNA against SARS-CoV-2 positive tests. In the analysis, the results are presented both in terms of the specimen collection date (not available in real-time) and publicly reported date for tests (available in real-time) and demonstrate that there is a bigger lead time between wastewater and tests by report date than tests by specimen date. As tests by report date are complete and would be used in real-time, highlighting this difference is useful to know.

In the example of Grover and colleagues [26], care is taken to explicitly state how the retrospective analysis was carried out using data that was revised, greatly helping the reader understand how the work could be applied in real time. Where the true unrevised data was unavailable, the authors simulated what would have been available, for example, with wastewater data they removed the most recent 4 days to account for the processing time involved.

Where available, data should be retrospectively analysed in its real-time available version, rather than finalised only, giving the most representative estimate of lead times. Data latency for indicator and signal should be clearly reported, alongside the extent of real-time to final data revisions. Using this information in downstream analysis can help establish an operational leading relationship between indicator and signal.

### 4. Single or multiple events

*Researchers should articulate whether a subject can influence the indicator or signal once or at multiple time points.*

Infections and their outcomes can be measured in many ways. At an individual level, infection events are defined to occur at a point in time, such as the onset of symptoms, but other states can be observed for an interval of time, such as being a person being symptomatic. More mechanistically, an individual generally enters a state once at a fixed point but can stay in that state for multiple time points. These pairs of events and states are common in epidemiology: symptom onset and being symptomatic [58], hospital admission and being in hospital [59], infection incidence and prevalence [60].

In an event-state pair, the state is defined by the event time and duration of individual in the state. At an individual level, the state is a consequence of the event; in aggregate this causes the appearance of leading relationships between counts of events and counts of individuals in a state. If the duration of state is trivially short, then the event and state will be matched in time. If the time duration of state is long, then an estimated lead time from event to state will be directly related to the duration of the state. This relationship between event and state time series can be defined by a mathematical convolution [27,61]. Rather than an indicator and signal; an event-state relationship is more akin to a signal and a transformed version of that same signal—which has limited real-world utility. It is crucial that researchers express whether their indicators and signals are measures of events and/or states. By conflating the two, conclusions about leading indicator relationships can be misleading.

Furthermore, individuals may also appear across multiple data sets which complicates the relationship between indicator and signal. For example, an infected individual could go to a pharmacy which could be detected, and when their symptoms deteriorate, they may attend primary or secondary care where again their infection would be recorded.

In their paper exploring the relationship between influenza hospital admissions and bed occupancy peaks, Wood explicitly models the relationship between the two data as a convolution [27]. This helps demonstrate that the lead time of hospital admissions is an artefact of how hospitalised patients are measured, rather than a unique signal.

In the work of D’Auost and colleagues [28], a range of SARS-CoV-2 surveillance metrics are compared. Wastewater viral RNA (each individual influences the signal at multiple time points, influenced by the duration of viral shedding) is compared with case counts and positivity (each individual is measured once). Furthermore, these are then compared with hospital beds occupied (where patients are measured at multiple time points, influenced by the patient duration of stay). As a result, the leading relationships found will be sensitive to the patient length of stay and duration of viral shedding, rather than unique information in the time series.

## Recommendations: Analysis

### 5. Temporal and spatial granularity

*Researchers should avoid generating unmeasured data and prioritise the temporal and spatial scale of the signal.*

Non-traditional surveillance data are often not measured at consistent granularities, which makes comparisons challenging. Temporally, we may see daily, weekly, or monthly data, and spatially there may be data at the level of administrative region, county, or nation [62]. Furthermore, some data may not have clearly defined geographical bounds, such as hospital catchments [63] or geographies not typically used in epidemiology, such as Google Trends use of “Designated Market Areas” areas [64]. The preferred temporal and spatial granularity should be that of the signal, rather than the indicator, as it is the trend used for decision-making.

The mapping from one granularity to another can cause loss in information, for example, by converting the indicator from daily to weekly aggregation to align with the signal [65,66]. Aggregating granularities is preferred to the alternative, which would require estimating the weekly signal as daily data, in essence estimating data that has not been observed. As with smoothing (Recommendation 7), interpolation can cause unrealistically optimistic associations to be made that may not be knowable in real-time. In cases where one granularity is not strictly a subset of another, such as different administrative geographies, then a common coarser area should be considered.

Within Shi and colleagues [29], exploring dengue forecast modelling, the authors clearly state how the data granularities change which is useful for the reader to understand. The authors explicitly state that daily covariates are coarsened to weekly to match the granularity of the signal in question, case data.

The temporal granularity of an indicator can be particularly challenging in the context of wastewater, where data may not be collected every day. In the work of Armenta-Castro and colleagues, the authors measure wastewater data on a Monday each week, then use that value as a weekly estimate, compared to a weekly average of daily SARS-CoV-2 positive cases [30].

Spatial and temporal granularities are important as they help uncover local and changing heterogeneities in the indicator and signal relationships. This trend heterogeneity in different areas, populations and time periods can strengthen confidence in indicator-signal relationships. However, care must be taken to avoid generating unrealistic data via interpolation and prioritise the granularity of the decision-making signal.

### 6. Time-varying relationships

*Researchers should present the evaluation over multiple time periods to demonstrate if leading relationships are consistent.*

The relationship between indicator and signal can vary over time due to sampling biases (opportunistic sampling) [67], changing biological systems (vaccination, strains, immunity) [68], control strategies [69], and other factors. Assuming a fixed relationship over a study period produces an average effect, hiding the changing nature of the relationship. Understanding the underlying mechanism between the leading indicator and signal will help to highlight how rapidly time-varying relationships are expected to change (Recommendation 1). For example, with SARS-CoV-2, there could be changes to variants every few months [70,71]. Alternatively, for more endemic pathogens where strains change [72], treating longer periods like a year separately may be appropriate. Using a single average effect may be convenient for reporting but hides potentially high utility periods or situations where the leading indicator loses effectiveness.

A simple approach to exploring the time-varying lead effect would be to separate the time period of the study into contiguous time ranges, such as 3-month durations, and analyse the relationship separately. If the researcher’s mechanistic understanding of the data indicates other periods may be distinct (such as periods of interventions, strain replacement, testing policies) then these could be used. Some methods, such as dynamic time warping [73], incorporate this time-varying effect into their approach explicitly, allowing for a varying relationship between indicator and signal to be demonstrated [74]. Alternatively, rolling windows of smaller time steps could be analysed one by one, showing the change in leading relationship over time.

Anderson and colleagues [31] perform a temporal analysis of RSV (Respiratory Syncytial Virus) surveillance indicators over multiple seasons. The authors produce analysis separated by season as well as assessing cross-correlation over a rolling 26-week window, stratified by pre-, during- and post-pandemic eras. This approach gives a strong expression of the variation in relationship between data sources over time.

In their analysis of Google Flu Trends with influenza-like-illness surveillance, Cook and colleagues [32] segment the periods around the 2009 H1N1 pandemic to give a better understanding of how the indicator and signal relate. They include a pre-pH1N1 period, and then separate pH1N1 waves to show different model performance, as well as giving an overall pH1N1 score.

Time-varying relationships are important because they can impact decisions made in real time, for example, by assuming a constant relationship between signal when the relationship is changing over time one might observe a more optimistic view of an epidemic trend. A rapidly changing relationship between signal and indicator can impact the performance of forecast models, leading to poor prospective model performance [8]. Care should be taken in identifying the size of time periods to analyse—shorter periods may be under-powered due to fewer data, while larger periods may average over changing effects. Crucially, all data should be used, rather than selecting only periods of high correspondence. Fig 4 demonstrates via simulation how the duration of a state can impact the lead timing between new hospitalisations and bed occupancy.

**Fig 4:**
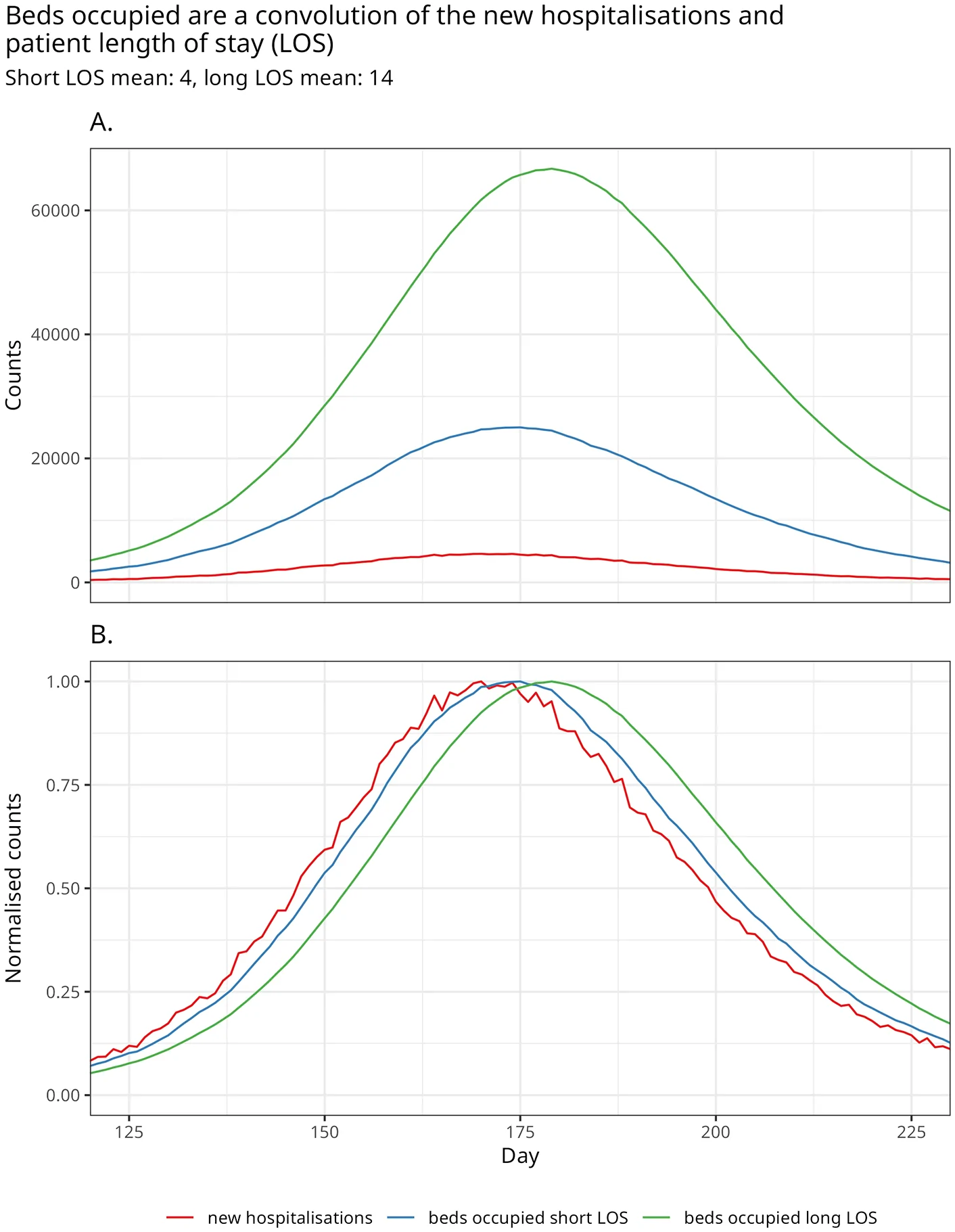
Using a simulation of epidemiological data, the effects event and state durations are explored. Taking new hospitalisations and convolving them over time with a (Gamma) length of stay (LOS) distribution to generate total beds occupied over time for short and long LOS. **(A)** shows the counts of new hospitalisations and beds occupied. Due to the length of stay there are many more currently occupied beds than new admissions each day. **(B)** shows a normalised time series (dividing by the maximum value per metric) to demonstrate the different average lags between new hospitalisations and beds occupied.

### 7. Smoothing and denoising

*If smoothing is necessary, researchers should choose a consistent method for avoiding the use of information unavailable in real-time.*

Epidemic time series data are often noisy and challenging to interpret, making smoothing an attractive choice. Data smoothing is used to adjust for statistical artefacts, such as day-of-week effects, mitigate the effect of outliers, make trends visually clearer and quantify uncertainty. Analysts performing leading indicator analyses frequently use rolling average methods [30,75–77]. For example, by using a 7-day rolling average on logarithmic-transformed daily data, the reporting effect of weekends (which may not be relevant to the analysis) can be reduced, allowing for easier comparison with other data as discussed in Bloom and colleagues’ work on indicator identification [16].

Crude approaches to smoothing, such as rolling averages can appear to visually reduce variation in data. However, rolling averages do not remove bias from estimates but rather combine bias from a range of consecutive points. Furthermore, uncertainty in the time series can be valuable to understand but is lost by averaging, as discussed in Aber and colleagues [78]. To produce less biased smoothed estimates of epidemic time series statistical models should be used, helping to understand the uncertainty associated with and across time series, such as using a Generalised Additive Model (GAM) with splines [12], random walk or Gaussian process [79] each with different underlying assumptions about the time series. These statistical methods can be used to remove artefacts such as day-of-week effects explicitly, as demonstrated in the syndromic surveillance analysis of Morbey and colleagues via a mixed-effect model [34] and quantify uncertainty in the time series.

A challenge with statistical methods for denoising surveillance data is that they can be sensitive to subjective parameter choice, such as knots in the case of a spline. Smoothing choices can drive different correlations between signal and indicator, demonstrated by You and colleagues [33], where different GAM methods produce negative or positive cross-correlations when a long-term trend is present. The optimal parameters for a smoothing model may not be known in real-time, and therefore retrospective results can naturally be more optimistic.

Real-time analysis should be considered with regards to smoothing methods. Smoothing methods are often applied to data sets with complete reporting after the epidemic. If we have a signal at time *T*, *y*(*T*), we want to produce a smoothed estimate $\hat{y}(T)=s(y(T))$. Smoothing methods leverage consecutive data to inform estimates, e.g., *y*(*T* + *x*). This is using “future” data to estimate values for today, which is only available in retrospective analysis, and can cause overly optimistic estimates for $\hat{y}$ due to “temporal leakage”. The difference between what is observed in real-time and in retrospect may be particularly important at crucial epidemic moments, such as at a peak. In the work of Eales and colleagues statistical methods are used to estimate respiratory case trends in Victoria in real-time but also compared to retrospective analysis [35]. They show that while the real-time estimates generally correspond to the retrospective, there can be differences in the two analyses. The comparison of both retrospective and real-time data helps decision makers have confidence in the real-time utility.

However, not all smoothing methods incorporate future data; a right-aligned rolling average only uses information *y*(*t* = 1), …, *y*(*t* = *T*) up to *T*, which is reproducible in real-time, though introduces other biases. The use of only data available in real-time is referred to as “causal filtering” [80]. When using a smoothing method to analyse leading indicators, it’s important to use the same method and preferably the same parameters to avoid introducing different temporal lags or biases into each time series estimate. The impact smoothing can have on timing is shown in Fig 5, with different methods producing differently timed epidemic indicators. The 21-day rolling average clearly lags the 7-day and LOESS (Locally Estimated Scatterplot Smoothing) smooth (Fig 5A). On the other hand, the two GAM models with different penalisation assumptions give similar, but different estimates of the time series (Fig 5B).

**Fig 5:**
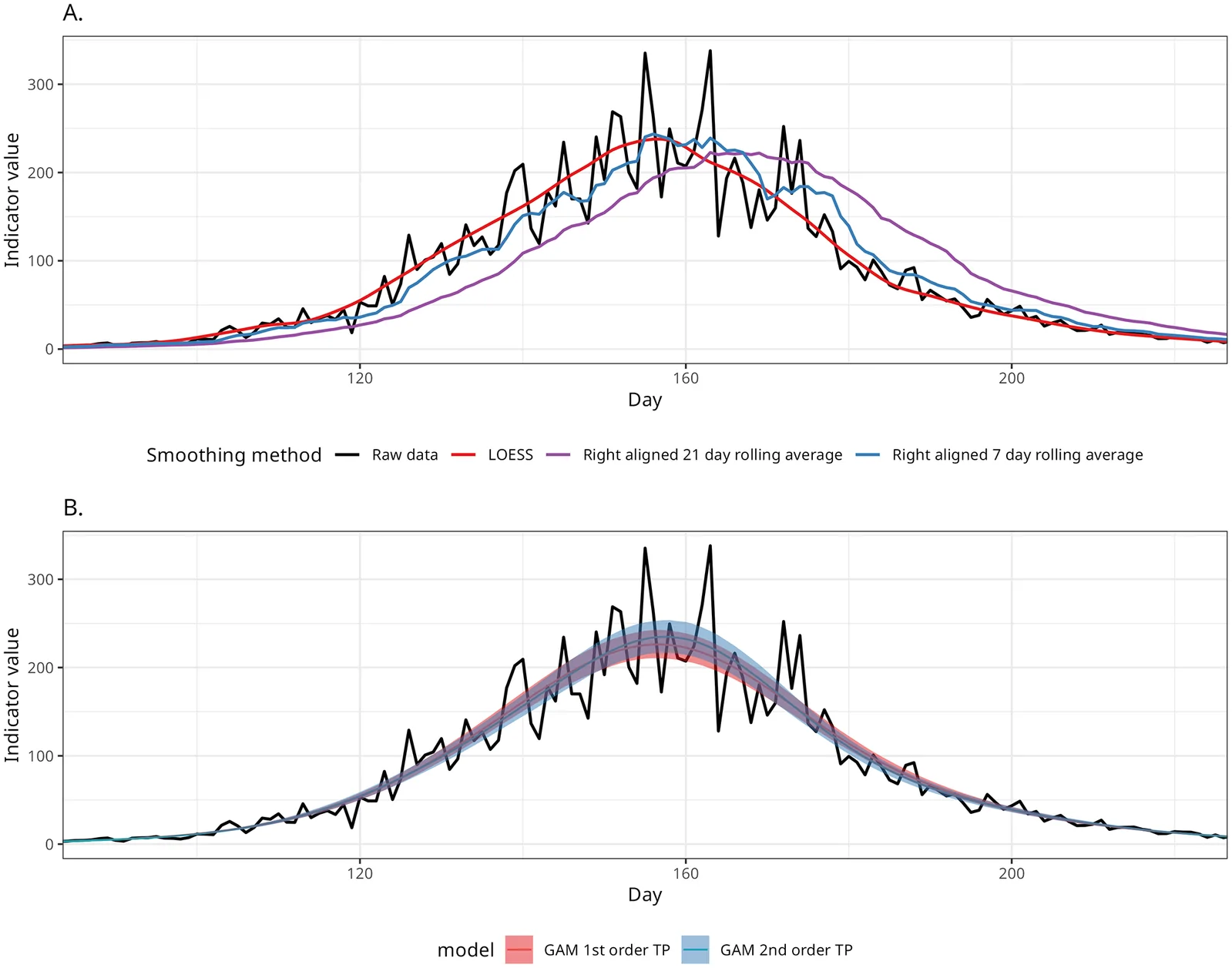
An indicator with a range of smoothing methods applied. **(A)** demonstrates LOESS and a right-aligned rolling average with two window lengths: 7 days and 21 days. The right-aligned method introduces a noticeable lag in the smoothed variables. The 7-day smooth is noisy relative to the 21-day version. **(B)** shows two GAM models fit to the indicator data with associated median estimate and 90% confidence intervals. The two models differ slightly, in that one Thin-Plate (TP) spline penalises the first-order derivative, the other the second-order.

Smoothing that is plausible in real-time, using only information available up to a given time point, can improve the interpretability of leading indicator analyses, but care must be taken to avoid using future information to improve the perceived leading relationship. The unsmoothed data may be more appropriate to report in the analysis, even if the indicator-signal relationship is not as strong, if unsmoothed data would be used by decision makers in real-time. Using smoothed and unsmoothed data in a sensitivity analysis helps demonstrate the robustness of the relationship. It is critically important that the methods and assumptions about the smoothing of data are clearly explained in the manuscript due to the extent they can impact downstream analysis.

### 8. Transformation: Logs, rates, scaling, magnitude and direction

*Researchers should mathematically transform their data appropriate to the infection process and sampling method of each data, and align the data with the decision-making process.*

Methods used to evaluate leading relationships may need certain properties from the data, such as linear relationships, stationarity, population normalisation or similar magnitudes, which may require transformation of the data. Furthermore, the signal may be transformed to be more useful for decision makers, such as the growth rate of cases rather than their counts [81]. The indicator and signal will frequently be different types of measurements and may capture different parts of the epidemic process. As a result, transformations on the data should be considered.

Researchers need to engage with decision makers to understand what metrics are useful in real-time [81], which will impact the transformations chosen. A common derived metric from surveillance is the epidemic growth rate [82], where the relative change over time is estimated. For methods that rely on assumptions of linearity between indicator and signal, then performing a logarithm of the indicator and signal may be appropriate. The choice of transformation should be driven by prior knowledge or theory, rather than deploying a range of methods to selectively choose one that maximises the leading relationship.

Furthermore, statistical models used to smooth epidemic time series frequently make assumptions on transformed scales which can differ from standard time series analysis. In Steyn and colleagues Bayesian P-splines are explored, as well as second-order random walks [36]. Penalising the first-order derivative on a log scale is analogous to penalising the growth rate, an attractive property for smoothing epidemics. Penalising the second-order log derivative therefore enforces smoothness on the growth rate. Researchers often implement smoothing on the reproduction number, such as in Sebastian and colleagues, who compare multiple methods: first-order random walks, Ornstein–Uhlenbeck processes, second-order random walks, integrated Brownian motion and Gaussian processes [83]. However, care must be taken when focussing on the real-time reproduction number for leading indicator analyses, as they are often not possible to estimate for novel or non-traditional data sources such as search trends or meteorological variables.

The work of Rø and colleagues provides an exemplar for comparing indicators and signals on transformed scales [17]. The researchers transform a via statistical modelling a wide range of incidence and prevalence surveillance indicators to the epidemic growth rate of SARS-CoV-2, then compare this metric to multiple signals. By working in the epidemic growth rate scale the disparate indicators are more comparable. Furthermore, in McGough and colleagues [37], a range of transformations including the logarithm and square root are used to compare novel Zika indicators with traditional surveillance metrics, helping to understand the relationship between signal and indicator.

An example of the effect of transformations on analysis is shown in Fig 6, where different cross-correlation shapes are created by comparing: no transformation, smoothing, smooth logged data and the estimated growth rate.

**Fig 6:**
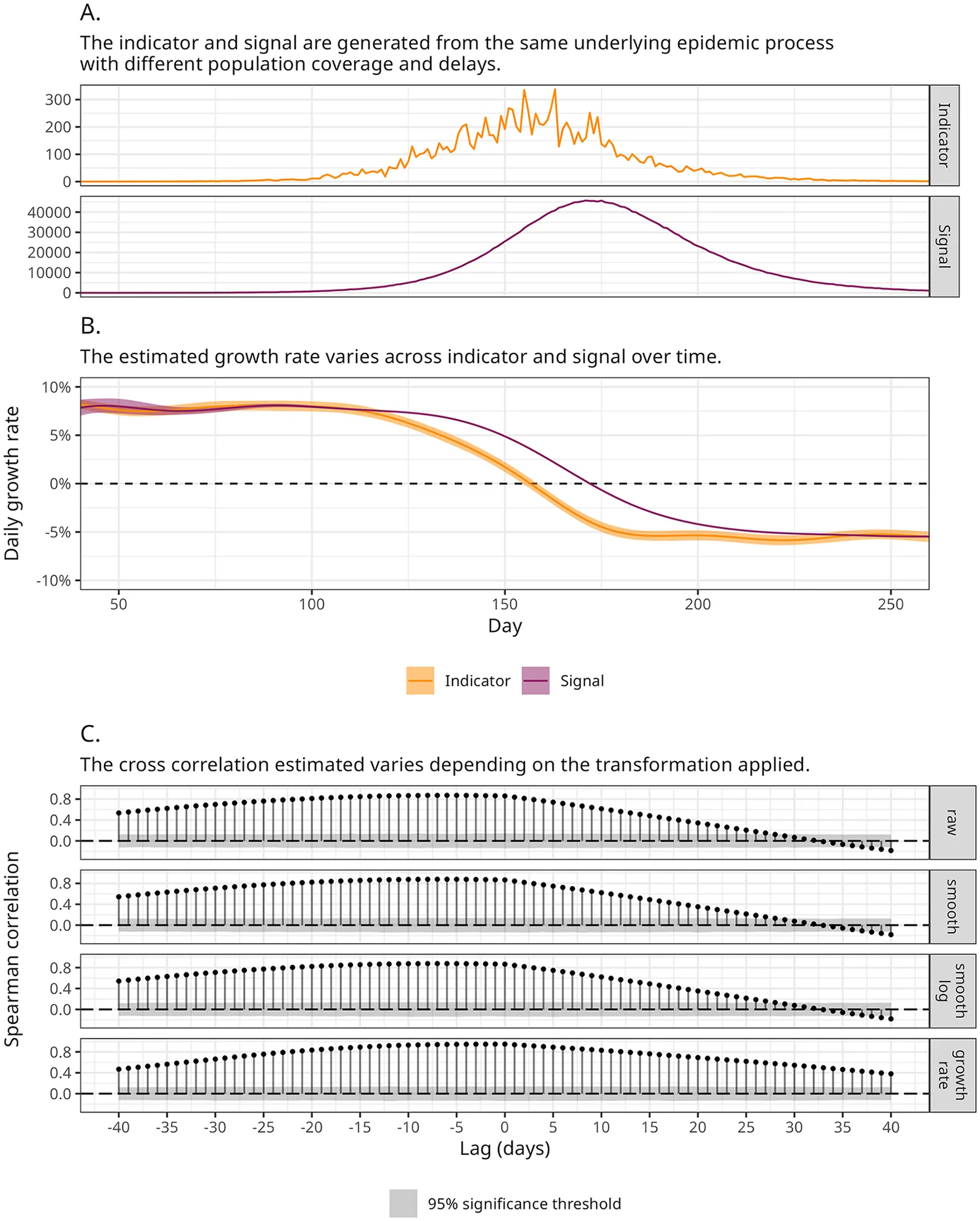
An example leading indicator analysis cross-correlation with bootstrap that is sensitive to transformation. **(A)** shows the raw indicator and signal over time. **(B)** shows the estimated growth rate using statistical modelling. **(C)** demonstrates the cross-correlation across transformations of the data. Spearman correlation was used to account for the non-linearity present in the time series. There is a clear leading relationship on the natural scale as there are generally higher correlations at negative lags. This is also the case for the smooth and smooth log transformations. The growth has a positive leading relationship, but it is less pronounced than the other transformation. The “indicator” is a young-age-biased incidence proxy (such as internet search trends), and the “signal” is reported cases in the elderly.

### 9. Uncertainty

*Researchers should propagate uncertainty through their analyses to conclusions about temporal relationships.*

Inferring a leading relationship between indicator and signal is inherently uncertain. There are many sources of variation when comparing the time series in question. These include uncertainty in the:

- observational noise of the indicator and signal time series
- method used for inference or estimation of leading relationships
- the underlying mechanism relating indicator to signal

In the literature, full joint modelling of indicator and signal that propagate uncertainty fulling in a system is rarely performed. However, the analyst should consider what the key sources of uncertainty in their analysis are and present these, rather than generating point estimates alone—which is common practice at present.

Uncertainty can be quantified in epidemic time series analysis in a range of ways. These include but are not limited to explicitly statistically modelling the time series for smoothing [17], bootstrapping to generate uncertainty in correlation estimates [84], providing a range of values when varying temporal period or location [85], distributed lag non-linear modelling [25,77].

In Ammerman and colleagues [39], the authors compare norovirus wastewater data to Google Trends, emergency department visits and school absences via cross-correlation. The lead times identified for each signal vary by spatial scale (zip code, county, state), which helps a user understand potential variation in practice.

Furthermore, conceptual uncertainty exists in the estimation of lead time estimation for epidemics. In Kumar and colleagues [18], the authors compile wastewater lead times across a wide range of global studies and show due to methodological choices and local variation studies, and lead times are not directly comparable. Going further, in Olesen and colleagues [86] the authors highlight that the definition of “leading indicator” itself is uncertain and varies across seemingly comparable studies substantially.

The inclusion of uncertainty in analysis of leading indicator relationships helps more appropriately communicate to users the confidence they should have in the leading indicator relationship. At an individual study level uncertainty in correlation is often demonstrated, but not commonly in estimated lead time – where point estimates are usually stated. An exception to this is the work of Asplin and colleagues [38], who provide prediction intervals in the estimated lag with maximum correlation values when comparing Google Trends to symptom positivity from a community symptom surveillance study.

In Fig 7 the leading relationship between indicator and signal are explored in terms of the peak timing. The indicator peaks earlier than the signal, however, analysing raw data may be sensitive to noisy observations rather than true trends. By applying a statistical model, we can estimate the lead time between the two peaks while also expressing the uncertainty in the estimate. Furthermore, using two different models gives us confidence that our conclusions are robust.

**Fig 7:**
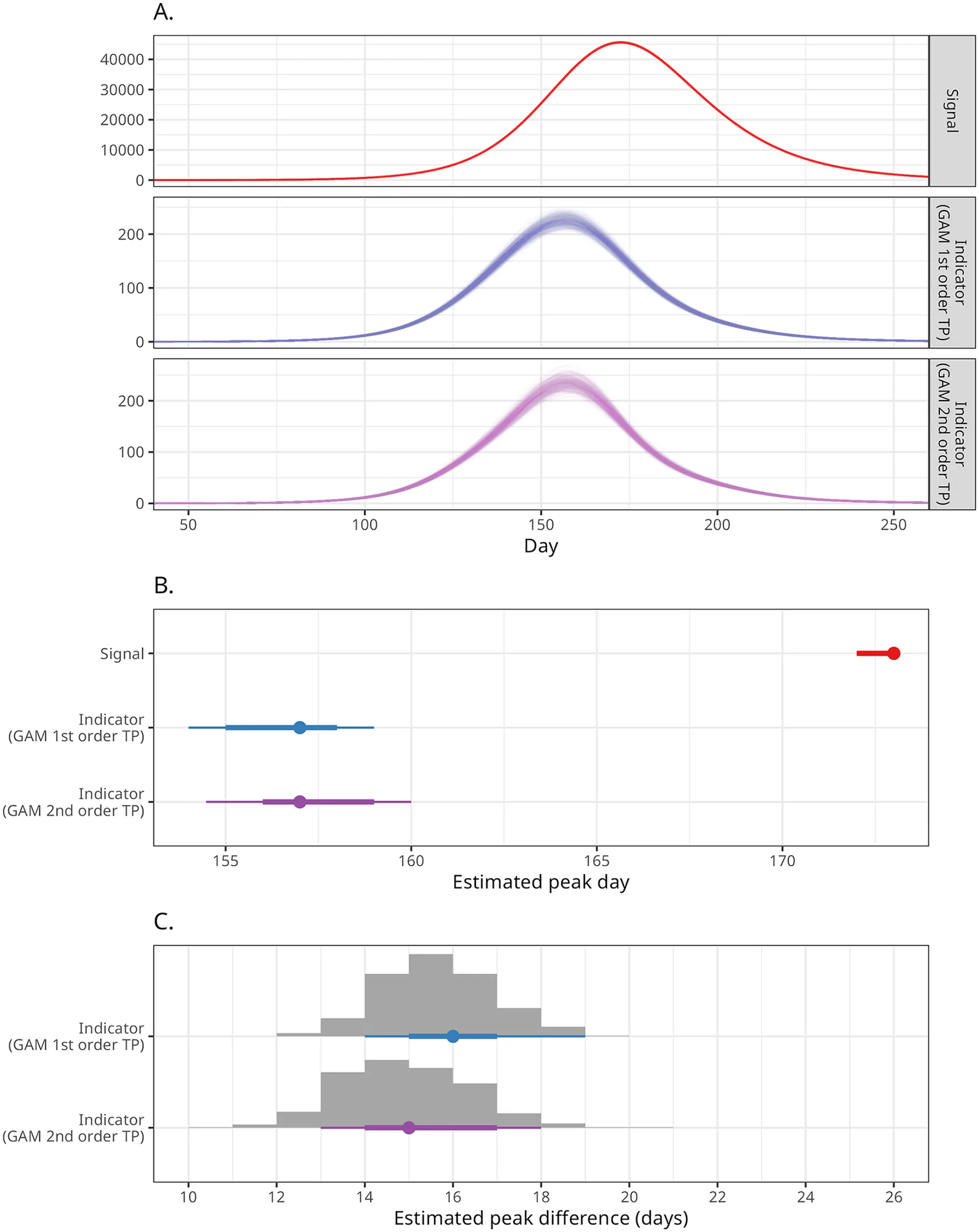
Demonstrates an analysis of the lead time between indicator and signal peak times using statistical models capturing uncertainty. The indicator and signal are the same as used in Fig 6. Two different models are presented for the indicator demonstrating that results are sensitive to model choices. First- and second-order thin-plate splines are used in the GAMs. **(A)** shows the modelled indicator and signal waves, where sample trajectories are presented. **(B)** shows the estimated peak timing for the indicator and signal with median, 66% and 95% intervals. **(C)** shows the difference in peak timing (a form of lead time) between indicator and signal with median, 66% and 95% intervals.

### 10. Predictive utility out of sample and baselining

*Researchers should demonstrate indicator lead times through predictive modelling of the signal and compare to a baseline.*

While methods that show the correlation or association between two signals are useful, often the intended purpose of an indicator is to directly predict (through a model or a decision maker’s head) the future values of the signal [87]. This is a verifiable statistical problem, whether the inclusion of an indicator in a model improves the predictions of the signal at some future time horizon, which helps to establish a predictive leading relationship. Furthermore, a leading relationship of X days, or an arbitrary correlation coefficient is not inherently useful—a baseline signal for analysis can be used to benchmark analysis against.

Epidemic signals are generally autocorrelated, due to the onward infection process [88]. As such, autoregressive models relying on the signal alone can be compared to the same model which also includes the indicator. By doing this, the predictive improvement of using the indicator can directly be quantified.

There are numerous examples of leading indicators being assessed using predictive modelling, such as Reich and colleagues [8] who explore the use of COVID-19 case reports to improve hospitalisation predictions. The authors show that inclusion of cases does not consistently improve predictions and are impacted by assumptions about reporting dates. McDonald and colleagues [40] start with a baseline autoregressive model, then augment the model using five different indicators to forecast COVID-19 case signals. Comparing the difference between the baseline model and inclusion of indicators allows the authors to explicitly conclude medical insurance claims, online symptom surveys and Google searches improve predictive performance.

Evaluating a leading indicator by showing improved predictive performance is a strong evidence base for a leading relationship. It is crucial that this is done using out-of-sample evaluation, where models are fit to a training period, and the performance is measured on a separate evaluation set of data to avoid overfitting. The choice of training and evaluation period is important—rolling and expanding forecast origins experimental design helps demonstrate a realistic situation to forecasts used in real-time. Using only data that would have been available in real-time (relating to Recommendation 3), including incompleteness and reporting delays. Accounting for these real-time factors helps to establish an operational lead, which is easier to translate to public health practice than less realistic evaluations. The EPIFORGE guidelines help analysts produce robust epidemic forecasting research [89]. Furthermore, the use of proper scoring rules in evaluating forecast performance allows for robust assessment of improvements to the data in an honest manner [90].

## Consequences

Each of the ten recommendations can impact the quality of leading indicator analyses. In Table 2, we outline the possible consequences of not considering each recommendation for decision making in real-time.

**Table 2:** The consequences of not following each recommendation relating to the operational use of leading indicators.

Type	Number	Name	Consequence if not followed
Reporting	1	Reporting of causal mechanisms	Associations between signal and indicator may be spurious and unrelated to infection dynamics, leading to incorrect poorly timed interventions in real-time.
	2	Sample coverage and bias	Associations identified between signal and indicator may be unknowingly influenced by factors that impact population subgroups differently, which may cause reduced lead times.
	3	Reporting delays and data revisions	Lead times identified retrospectively may be heavily reduced when indicators are used operationally reducing the timeliness of interventions.
	4	Single or multiple events	Lead times identified may be artefacts of measurement rather than due to unique information, reducing the utility of triangulating data for decision makers.
Analysis	5	Temporal and spatial granularity	Using indicators in real-time will be practically harder to do without appropriate spatiotemporal mapping, and performance may be lower in practice reducing decision-maker confidence in the analysis.
	6	Time varying relationships and lags	Decisions may be based on average relationships which may not represent reality causing poorly timed interventions.
	7	Smoothing and denoising	Different methods for smoothing may introduce artificial leads which will not replicate in real-time, and the use of future information will indicate overly optimistic relationships, reducing utility for decision advantages.
	8	Transformation: logs, rates, scaling, magnitude and direction	Strong lead relationships may be missed without the use of mechanism-informed transformations and relationships identified may not align with how the indicator will be used by decision makers.
	8	Transformation: logs, rates, scaling, magnitude and direction	Strong lead relationships may be missed without the use of mechanism-informed transformations and relationships identified may not align with how the indicator will be used by decision makers.
	9	Uncertainty	Using only point estimates in analyses hides inherent variation in relationships and implies a level of confidence that the data may not support.
	10	Predictive utility out of sample and baselining	Leading relationships may be artefacts of the statistical analyses conducted, rather than practically useful. Without a baseline to compare to, the usefulness of the lead time is not straightforward to assess. Without this, decision makers may have false confidence in indicators, reducing decision quality.

## Discussion

Robust surveillance of infectious disease is a key requirement of good decision-making during epidemics. Understanding how different surveillance systems relate and may predict one another in real time can provide an information edge that improves decisions. However, soundly demonstrating a leading relationship between indicator and signal is challenging retrospectively. There are a range of pitfalls which undermine the credibility of these types of analyses—by accounting for effects discussed in this recommendation list, researchers can improve the robustness of their conclusions for decision makers.

Leading indicator analysis and related work can be valuable for operational use, helping decision makers understand the current state of an epidemic, and improve the performance of important forecasting models. However, poorly considered leading indicator analyses can be detrimental to model performance and decision-making—introducing more confusion rather than clarification. Poor reporting in these analyses makes the research harder to replicate for operational public health teams, and harder to trust the analysis conducted. Without well describing operational challenges, such as latency and revisions, an overly optimistic picture of an indicator is created by researchers, which may be disappointing in real-time. Furthermore, conducting statistical analyses that do not consider the real-time dynamics and measurement can inflate indicator performance, making the benefits of an indicator challenging to replicate in practice.

We have focussed on making recommendations that are generalised to a wide range of surveillance indicators and statistical methods—but specific to real-time challenges. The shape, cadence and challenges associated with new surveillance indicators are myriad and cannot all be discussed, but the principles of the recommendations here should be considered at the start of analyses. There remains substantial room in the literature for improving methods for the identification and quantification of leading indicator relationships.

The use of statistical modelling when analysing leading indicators is highly desirable and facilitates the some of the guidance presented in this work. The quantification of uncertainty, adjustment for surveillance biases, smoothing and use of predictive modelling can be more easily achieved by applying statistical modelling to leading indicator analyses—improving the robustness of analyses. In many cases in the literature statistical models are not applied, which implies either the value of modelling is not believed in by some analysts, or there are higher complexities and difficulty associated with more advanced statistical techniques. Ensuring specific methods are easy to use for epidemiological time series analysis should be a priority for researchers to improve practices in the field.

There is substantial prior work on epidemic leading indicator analyses, both exploring indicators themselves and methodologies to identify them [6]. Work by Bloom and colleagues [16] highlights key caveats with a commonly used approach, the cross-correlation function, which has informed many analyses. Furthermore, the SARS-CoV-2 pandemic has driven improvements in this area [17], as well as highlighted room for improvement in surveillance and analyses [91].

This work is broad guidance exploring practices in the field of epidemic time series leading indicator analysis. Examples were not found via systematic review and therefore there will be some biases towards the literature discussed. The examples presented were generated via simulation to demonstrate specific reporting or methodological points, rather than using real-world data. Furthermore, we have not quantified how prolific the methodological challenges are in the wider literature, instead focussing on examples.

Throughout this work, we have highlighted the importance of centring the decision maker in analytical decisions. This is an important part of ensuring work is impactful, however, we recognise it is not always possible in research settings. Efforts should be made to identify users and failing that, researchers should consult literature for similar problems to the ones they face and have other analysts review their user-based assumptions. The works by Hadley and colleagues (2025, 2026) [92,93] explore the interface between researchers and policy decision makers which provides helpful context for researchers. In addition, Morgan [94] provides a guide as to how decision makers harness quantitative data to inform choices in outbreaks, which can help analysts inform their work.

A strong analysis of leading indicator relationships requires knowledge in a range of areas: epidemiology, statistics and real-time data analysis—a research team should cover these areas of expertise. This work will hopefully support improvements in practices for these analyses to avoid common pitfalls to help drive better real-time public health decision-making.

### Ethics statement

No ethical approval was required for this methodology-focused research using only simulated data.

## Supporting information

S1 Appendix Supplementary figure and tables. (DOCX)
