# Supplementary material for: Recommendations for the analysis of leading indicators in epidemic surveillance
Source: PLOS Digit Health. 2026 Oct 5;5(10):e0001759. doi: 10.1371/journal.pdig.0001759 (PMC13637922; doi:10.1371/journal.pdig.0001759)
Supplement: Supplementary file 1 [file pdig.0001759.s001.docx]

# Supplementary Information: Recommendations for the Analysis of Leading Indicators in Epidemic Surveillance

## Section 1: Data Simulation Protocol

Throughout the manuscript we provide simulated examples of infections and surveillance to demonstrate key recommendation concepts. In this section we outline how this data was generated.

**1.1 Infection model**

We use the default model from the *epidemics* R package [1], an SEIR-V deterministic compartmental model with age stratification (we do not incorporate vaccination in this case). The population of the UK is assumed, with a contact matrix derived from POLYMOD [2]. We use the following age groups to stratify the population and account for some differential mixing: [0,20), [20,40), [40,Inf). Compartment values are post-processed to integer values. Parameter values are given in Table S1. Incidence (new infections) are calculated as the number of individuals leaving the susceptible compartment at each time step, $I_{g}(t$) where $I$ denotes incidence, $g$ some subgroup and $t$ time. The incidence values over time are shown in Figure S1.

| **Parameter name** | **Value** |
| --- | --- |
| Total time | 300 |
| Transmission rate | 1.8 / 7 |
| Infectiousness rate | 1 / 2 |
| Recovery rate | 1 / 7 |

*Table A. Parameters of infection model.*

Fig A. Plot of new infections over time for each demography group in the infection model.

**1.2 Reporting delays and backfilling**

To explore delays in reporting we move from incidence to cases. This is done in two steps: 1) scale and 2) delay.

To transform the scale, we assume on average some fraction of incident infections is ascertained $\delta_{g}$ (the infection-case rate), with Poisson noise. Cases $C_{g}(t)$ are given by

$$C_{g}\left( t \right)=\mathrm{Poission}\left( {\delta_{g}I}_{g}\left( t \right) \right).$$

We add a delay between the infection event and a case being identified via a random variable $D_{\mathrm{identify}}$

$$D_{\mathrm{identify}} \sim\lfloor\mathrm{Gamma}(\alpha_{\mathrm{identify},}\gamma_{\mathrm{identify}})\rfloor$$

Giving for each case a delay $d_{\mathrm{identify}}$. We have the number of cases by infection date and reporting delay $c_{g}(t, d_{\mathrm{identify}})$, and can get the number of identified cases per day from

$$C_{g}\left( t_{\mathrm{identify}} \right)= \sum_{t=0}^{t=t_{\mathrm{identify}}} c_{g}(t, t_{\mathrm{identify}}-t)$$

We add an assumed reporting delay random variable $D_{\mathrm{report}}$ from identification to report at an individual level generated from a floored Gamma distribution with shape $\alpha_{\mathrm{delay}}$ and rate $\gamma_{\mathrm{delay}}$

$$D_{\mathrm{report}} \sim\lfloor\mathrm{Gamma}(\alpha_{\mathrm{delay},}\gamma_{\mathrm{delay}})\rfloor$$

which allows us to express a 2 x 2 array of cases by report delay $c_{g}(t_{\mathrm{identify}},d_{\mathrm{report}})$ such that

$C_{g}\left( t_{\mathrm{identify}} \right)=\sum_{d=0}^{d=d_{identify,max}} c_{g}(t_{\mathrm{identify}},d_{\mathrm{report}})$.

Because of this reporting delay in real-time the value of $C_{g}(t)$ is incomplete when $t<T-d$, where $T$ is the maximum time point available, a right truncation.

We can however aggregate by report date to get complete data, although impacted by the reporting delay observed. In this case we define $t_{\mathrm{report}}=t+d_{\mathrm{report}}$

$$C_{g}\left( t_{\mathrm{report}} \right)= \sum_{t_{\mathrm{identify}}=0}^{t_{\mathrm{identify}}=t_{\mathrm{report}}} c_{g}(t_{\mathrm{identify}},t_{\mathrm{report}}-t_{\mathrm{identify}})$$

and such we can compare $C_{g}(t_{\mathrm{identify}})$ and $C_{g}(t_{\mathrm{report}})$ over time. The parameter values for the simulation are given in Table S2.

| **Parameter name** | **Value** |
| --- | --- |
| $\delta_{g=[0,20)}$ | 0 |
| $\delta_{g=[20,40)}$ | 0 |
| $\delta_{g=[40,\mathrm{Inf})}$ | 0.2 |
| $\alpha_{\mathrm{identify}}$ | 4 |
| $\gamma_{\mathrm{identify}}$ | 1 |
| $\alpha_{\mathrm{report}}$ | 5 |
| $\gamma_{\mathrm{report}}$ | 1 |

*Table B. Parameters of reporting delays and backfilling.*

**1.3 Single and multiple events**

To explore the relationship between an event time series and state time series we simulate new hospitalisations and beds occupied. As with cases, hospitalisations are generated by transforming the incidence time series in terms of scale and delay.

To transform the scale, we assume on average some fraction of incident infections are hospitalised $\omega_{g}$ (the infection-hospitalisation rate), with Poisson noise. New hospitalisations $H_{g}(t)$ are given by

$$H_{g}\left( t \right)=\mathrm{Poission}\left( {\omega_{g}I}_{g}\left( t \right) \right).$$

We add a delay between the infection event and a hospitalisation being identified via a random variable $D_{\mathrm{hosp}}$

$$D_{\mathrm{hosp}} \sim\lfloor\mathrm{Gamma}(\alpha_{\mathrm{hosp},}\gamma_{\mathrm{hosp}})\rfloor$$

Giving for each new hospitalisation a delay $d_{\mathrm{hosp}}$. We have the number of new hospitalisations by infection date and reporting delay $h_{g}(t, d_{\mathrm{hosp}})$, and can get the number of new hospitalisations per day from

$$H_{g}\left( t_{\mathrm{hosp}} \right)= \sum_{t=0}^{t=t_{\mathrm{hosp}}} h_{g}(t, t_{\mathrm{hosp}}-t)$$

Given our new hospitalisations by admission date $H_{g}(t_{\mathrm{hosp}})$ we can now estimate the number of beds occupied via convolution. We define a length of stay probability mass function by discretising a Gamma distribution using the differenced cumulative distribution function, with some maximum delay considered $b_{\max}$.

$$D_{\mathrm{LOS}}\sim DiscreteGamma(\alpha_{\mathrm{LOS}}, \gamma_{\mathrm{LOS}})$$

Our distribution can therefore be defined by our probability mass function $d_{\mathrm{LOS}}(b)$ where $b$ is the days since admission of a patent and $d_{\mathrm{LOS}}$ is an array of probabilities for $b\in1, \ldots, b_{\max}$. In contrast to the reporting cases data triangle, we demonstrate an alternative but equivalent approach to generating a 2-dimensional array with a delay distribution (avoiding simulation at an individual level).

Using the discretised LOS distribution, we can sample from a multinomial distribution to generate the number of individuals $o_{g}$ admitted on a given $t_{\mathrm{hosp}}$ with a LOS duration $b$.

$$o_{g}\left( t_{\mathrm{hosp}},b \right)=Multinomial(H_{g}, b_{\max}, d_{\mathrm{LOS}})$$

The number of trials is determined by $H_{g}$, the number of mutually exclusive events $b_{\max}$ and the event probabilities $d_{\mathrm{LOS}}$. The total number of beds occupied over time $O_{g}\left( t_{\mathrm{hosp}} \right)$ is calculated as the sum of individuals currently hospitalised. We have the time of admission defined by $t_{\mathrm{hosp}}$ and the time of discharge is then $t_{\mathrm{discharge}}= t_{\mathrm{hosp}}+b$. We use an indicator function $A\left( t_{\mathrm{hosp}},b \right)$ which is non-zero when a patient is between admission and discharge time, to give us the count of occupied beds $O_{g}\left( t \right)$.

$$A\left( t_{\mathrm{hosp}},b \right)=\left\{ \begin{aligned} 0, &t<t_{\mathrm{hosp}} \\ 1, &t_{\mathrm{hosp}}\leq t\leq t_{\mathrm{hosp}}+b \\ 0, &t\geq t_{\mathrm{hosp}}+b \end{aligned} \right.$$

$$O_{g}\left( t \right)= \sum_{t=1}^{{t=t}_{\mathrm{hosp},\max}+b} \sum_{b=1}^{{b=b}_{\max}} A\left( t,b \right)*o_{g}(t,b)$$

We create two time series of beds occupied data by varying $\alpha_{\mathrm{LOS}}$. Under this formulation the beds occupied signal is a weighted sum of past new hospitalisations, influenced by the length of stay distribution.

| **Parameter name** | **Value** |
| --- | --- |
| $\omega_{g=[0,20)}$ | 0 |
| $\omega_{g=[20,40)}$ | 0 |
| $\omega_{g=[40,\mathrm{Inf})}$ | 0.02 |
| $\alpha_{\mathrm{hosp}}$ | 7 |
| $\gamma_{\mathrm{hosp}}$ | 1 |
| $\alpha_{LOS,short}$ | 4 |
| $\gamma_{LOS,short}$ | 1 |
| $\alpha_{LOS,long}$ | 14 |
| $\gamma_{LOS,long}$ | 1 |

*Table C. Parameters for new hospitalisation and bed occupancy convolution simulation.*

**1.4 Indicator**

To compare indicator to signal we need to generate the indicator. The indicator we choose is inspired by internet search volumes. We assume that the indicator timing is related to disease incidence – making it and “early indicator” of cases detected. First, we generate a weighting by age group of their influence on the indicator, a proxy for internet access or propensity to use online healthcare seeking behaviour. The weighting by demographic group is denoted as $\kappa_{g}$. Furthermore, our indicator is imperfect and subject to a random noise (it is an imperfect proxy). We take draws $\epsilon_{g}$ from $\mathrm{Gamma}(\alpha_{\mathrm{noise}},\beta_{\mathrm{noise}})$ for each demographic group, where $\alpha_{\mathrm{noise}}$ is the shape and $\beta_{\mathrm{noise}}$ the rate of our noise term. Our overall indicator $V(t)$ is calculated as

$$V\left( t \right)= \sum_{g} \frac{I_{g}\left( t \right) \kappa_{g} \epsilon_{g}}{1000}$$

which is a combined signal across all age groups because indicators like search terms do not typically provide demographic breakdowns. We divide by 1000 to produce a value on the different scale to cases. We force the resulting values to be non-negative to represent a volume proxy. The parameters used for this analysis are given in Table S4.

| **Parameter name** | **Value** |
| --- | --- |
| $\kappa_{g=[0,20)}$ | 0.7 |
| $\kappa_{g=[20,40)}$ | 0.2 |
| $\kappa_{g=[40,\mathrm{Inf})}$ | 0.1 |
| $\alpha_{\mathrm{noise}}$ | 10 |
| $\beta_{\mathrm{noise}}$ | 10 |

*Table D. Parameters of leading indicator generation.*

**1.5 Smoothing models**

To demonstrate statistical models used for smoothing in Figure 4 we fit two models to the indicator data. The models were fit using the R package (cite) *mgcv* with estimates generated using *gratia* (cite). We estimate $V(t)$ using two generalised additive models using thin-plate splines. $V(t)$ is assumed to be Gamma distributed with a log-link, with a shape-rate parameterisation.

$$V\left( t \right)\sim\mathrm{Gamma}(\alpha, \beta)$$

$$\mu=\frac{\alpha}{\beta}$$

GAM 1^st^ order TP: $\log\left( \mu\right)=V\left( t \right)=\beta_{0}+s_{1}\left( t \right)$

GAM 2^nd^ order TP: $\log\left( \mu\right)=V\left( t \right)=\beta_{0}+s_{2}\left( t \right)$

Where $s_{1}(t)$ is a first order penalised thin-plate spline, and $s_{2}(t)$ is a second order thin plate spline. Draws of the expected values are generated via Metropolis-Hastings and intervals created via quantiles. The median and 90% intervals are presented. The number of knots used for the smoother was given as $k=\mathrm{round}(\frac{t_{\max}}{20})$, giving one knot every 20 time points. Similarly, to smooth the reported cases we regress with a GAM, assuming cases follow a negative binomial error structure with a location-dispersion parameterisation.

$$C\left( t \right) \sim\mathrm{NegativeBinomial}(\mu,\phi)$$

$$\log\left( \mu\right)=\beta_{0}+s_{1}(1)$$

For this model we use the same first order thin plate spline, and $k$ as the indicator models.

For both the indicator and signal models we can estimate samples from the growth rate via finite differences of the linear predictor.

$$r\left( t \right)= \frac{d log(\mu)}{dt}$$

**1.6 Peak detection**

To demonstrate the uncertainty in leading indicator analysis we present a peak timing analysis between the leading indicator $V(t)$ and cases $C(t)$ previously described. We propagate uncertainty from the modelling into the final leading analysis calculation.

For the indicator we apply the analysis to both the GAM 1^st^ order TP and the GAM 2^nd^ order TP. For cases we use the previous negative binomial model with a first order thin-plate spline.

We draw 500 estimates of the indicator and signal, each indexed by $i$, denoted as $v_{i}(t)$ and $c_{i}(t)$. The peak timing for each trajectory are denoted by

$$t_{v,i,\mathrm{peak}}=\mathrm{argmax}_{t}(v_{i}\left( t \right))$$

$t_{c,i,\mathrm{peak}}=\mathrm{argmax}_{t}(c_{i}\left( t \right))$.

We can therefore explore the distributions $t_{v,i,\mathrm{peak}}$ and $t_{c,i,\mathrm{peak}}$ to identify with uncertainty the peak timing of the wave. Furthermore, matching modelled draws across the two models we can calculate

$$t_{i,\mathrm{lead}}=t_{c,i,\mathrm{peak}}-t_{v,i,\mathrm{peak}}$$

And explore the distribution of the peak timing lead time. We take summary statistics of $t_{v,i,\mathrm{peak}}$, $t_{c,i,\mathrm{peak}}$ and $t_{c,i,\mathrm{peak}}$ to communicate the epidemic peak lead timing between metrics.

## References

1.	Ahadzie, P.G.a.R.E.a.E.V.L.a.B. *epidemics: Composable Epidemic Scenario Modelling*. 2026; Available from: <https://github.com/epiverse-trace/epidemics>.

2.	Mossong, J., et al., *Social Contacts and Mixing Patterns Relevant to the Spread of Infectious Diseases.* PLOS Medicine, 2008. **5**(3): p. e74.
